# Annual Meeting of the Merimack Valley Dental Association

**Published:** 1865-11

**Authors:** 


ANNUAL MEETING OF THE MERRIMACK VALLEY DENTAL ASSOCIATION. — The third annual meeting of the Merrimack Valley Dental Association opened at the Phœnix Block, Concord, N. H., Nov. 2, at 11 o'clock A. M. There was a fair attendance, including dentists from Concord, Manchester, Franklin, Lebanon and Claremont in this State, and from Boston, Lowell, Lawrence, Milford and other places in Massachusetts. The presence of Prof. W. H. Atkinson, of the New York College of Dentistry was a source of much gratification to the members of the Association.

Soon after the meeting opened, the annual address was given by the President, Dr. Ambrose Lawrence, of Lowell. Subsequently the Association elected the following officers for the ensuing year; President, Dr. A. Lawrence; Vice Presidents, D. K. Boutelle of Manchester, and E. G. Cummings of Concord; Recording Secretary, G. A. Gerry of Lowell; Corresponding Secretary, J. H. Kidder of Lawrence; Treasurer, S. Lawrence of Lowell; Librarian, G. A. Gerry; Executive Committee, E. G. Cummings of Concord, S. L. Ward of Lowell, D. T. Porter of Lawrence, J. W. Little of Concord, and L. F. Locke of Nashua.

At the afternoon session Dr. J. H. Kidder read an interesting essay entitled "Mutual Relations of Dental and Medical Practitioners." The Association then entered upon the discussion of the subject of "Conservative Dentistry." Prof. Atkinson opened the debate with extended remarks, and was followed by Doctors Lawrence of Lowell, Salmon of Boston, Cook of Millford, Mass., Cummings of Concord, and others. The discussion proved very entertaining and instructive.

It was decided to hold the next annual meeting in Lowell. Dr. Moses T. Willard of this city has been appointed essayist.

The evening of Thursday was occupied by a discussion upon root filling when alveolar abscess had taken place. The remarks were mostly by Professor Atkinson. At the close of the session a social entertainment was given at the expense of Dr. Cummings in Phœnix Block. On Friday clinical demonstrations were given by Prof. Atkinson, and a communication read from Prof. J. H. McQuillen of the Philadelphia Dental College. The theme assigned for consideration at the next annual meeting is "Conservative Dentistry."

